# [(2*S*)-2-(3,5-Dichloro-2-oxidobenzyl­ideneamino)-3-(4-hydroxy­phen­yl)propionato-κ^3^ *O*,*N*,*O*′](dimethyl­formamide-κ*O*)copper(II)

**DOI:** 10.1107/S1600536808007939

**Published:** 2008-03-29

**Authors:** Ming-Xiong Tan, Zhen-Feng Chen, Zhou Neng, Hong Liang

**Affiliations:** a College of Chemistry and Chemical Engineering, Central South University, Changsha, HuNan 410083, People’s Republic of China; b Department of Chemistry and Biology, Yu Lin Normal College, Yulin, Guangxi 537000, People’s Republic of China; c Key Laboratory of Medicinal Chemical Resources and Molecular Engineering, Ministry of Education, College of Chemistry and Chemical Engineering, Guangxi Normal University, Yucai Road 15, Guilin 541004, People’s Republic of China

## Abstract

In the title complex, [Cu(C_16_H_11_Cl_2_NO_4_)(C_3_H_7_NO)] , the Cu^II^ atom is coordinated by two O atoms and one N atom from the tridentate ligand *L* ^2−^ {*L*H_2_ = (2*S*)-[2-(3,5-dichloro-2-hydroxy­benzyl­idene)­imino]-3-(4-hydroxy­phenyl)propionic acid} and one O atom from a dimethyl­formamide mol­ecule, resulting in a slightly distorted square-planar geometry. The structure forms a one-dimensional chain through weak coordination bonds [Cu⋯O 3.080 (1), Cu⋯Cl 3.269 (1) Å] and a three-dimensional network through O—H⋯O and C—H⋯O hydrogen bonds.

## Related literature

For related structures, see: Li *et al.* (2008 ▶); Zhang, Li *et al.* (2007 ▶); Zhang, Feng *et al.* (2007*a* ▶,*b* ▶). For related literature, see: Xia *et al.* (2007 ▶); Liu *et al.* (2007 ▶); Cohen *et al.* (1964 ▶); Desiraju (1989 ▶); Zordan *et al.* (2005 ▶). 
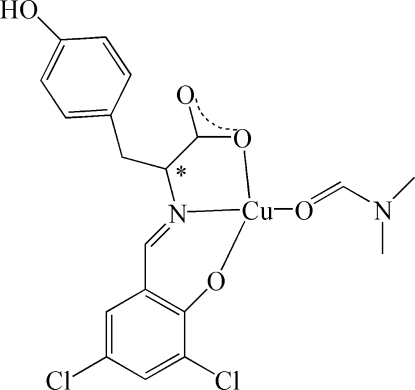


## Experimental

### 

#### Crystal data

[Cu(C_16_H_11_Cl_2_NO_4_)(C_3_H_7_NO)]
*M* *_r_* = 488.79
Orthorhombic, 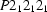

*a* = 5.8646 (16) Å
*b* = 13.220 (2) Å
*c* = 26.850 (3) Å
*V* = 2081.7 (7) Å^3^
*Z* = 4
Mo *K*α radiation
μ = 1.34 mm^−1^
*T* = 298 (2) K
0.48 × 0.20 × 0.18 mm

#### Data collection

Bruker SMART CCD area-detector diffractometer
Absorption correction: multi-scan (*SADABS*; Sheldrick, 1996 ▶) *T*_min_ = 0.532, *T*_max_ = 0.786
10650 measured reflections
3638 independent reflections
2915 reflections with *I* > 2σ(*I*)
*R*_int_ = 0.098

#### Refinement

*R*[*F* ^2^ > 2σ(*F* ^2^)] = 0.061
*wR*(*F* ^2^) = 0.148
*S* = 1.04
3638 reflections
262 parameters
H-atom parameters constrained
Δρ_max_ = 0.50 e Å^−3^
Δρ_min_ = −0.54 e Å^−3^
Absolute structure: Flack (1983 ▶), with 1505 Friedel pairs
Flack parameter: 0.04 (3)

### 

Data collection: *SMART* (Bruker, 2001 ▶); cell refinement: *SAINT* (Bruker, 2001 ▶); data reduction: *SAINT*; program(s) used to solve structure: *SHELXS97* (Sheldrick, 2008 ▶); program(s) used to refine structure: *SHELXL97* (Sheldrick, 2008 ▶); molecular graphics: *SHELXTL* (Sheldrick, 2008 ▶); software used to prepare material for publication: *SHELXTL*.

## Supplementary Material

Crystal structure: contains datablocks global, I. DOI: 10.1107/S1600536808007939/rt2016sup1.cif

Structure factors: contains datablocks I. DOI: 10.1107/S1600536808007939/rt2016Isup2.hkl

Additional supplementary materials:  crystallographic information; 3D view; checkCIF report

## Figures and Tables

**Table:** 

Cu1—O4	1.875 (5)
Cu1—O1	1.931 (5)
Cu1—N1	1.934 (5)
Cu1—O5	1.954 (4)

**Table:** 

O4—Cu1—O1	169.0 (2)
O4—Cu1—N1	94.2 (2)
O1—Cu1—N1	84.5 (2)
O4—Cu1—O5	90.2 (2)
O1—Cu1—O5	91.9 (2)
N1—Cu1—O5	174.3 (2)

**Table 2:** Hydrogen-bond geometry (Å, °)

*D*—H⋯*A*	*D*—H	H⋯*A*	*D*⋯*A*	*D*—H⋯*A*
O3—H3⋯O2^i^	0.82	1.87	2.661 (7)	163
C17—H17⋯O4	0.93	2.27	2.743 (8)	111
C18—H18*B*⋯O1^ii^	0.96	2.54	3.421 (10)	150

Symmetry codes: (i) 
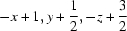
; (ii) 
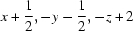
.

**Fig. 1.**
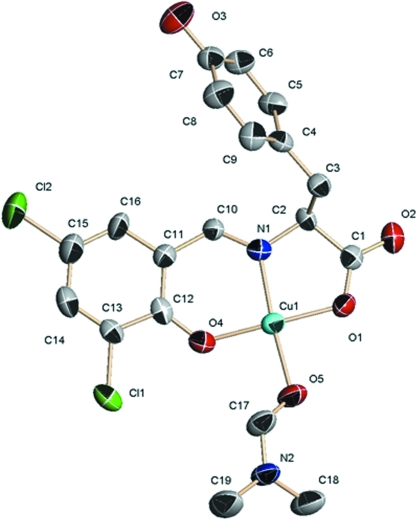
The molecular structure of (I), showing 30% probability displacement ellipsoids for non-H atoms. hydrogen atoms have been omitted.

**Fig. 2.**
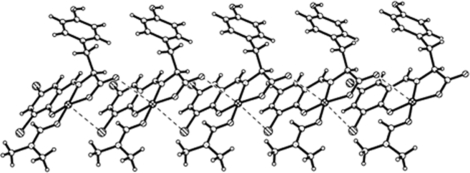
one-dimensional chain of (I).

**Fig. 3.**
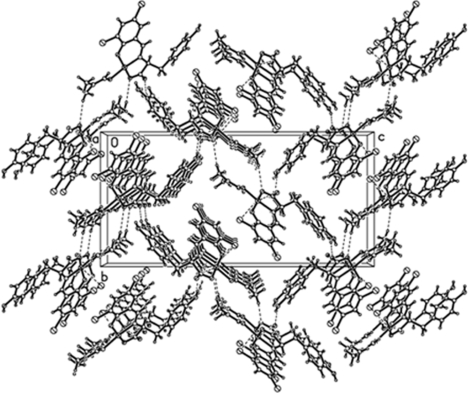
The three-dimensional network of (I) through hydrogen bonds.

## supplementary crystallographic information

### Comment

Halogens have a ubiquitous presence in both inorganic and organic chemistry, serving as mondentate or bridging ligands for a wide variety of d-block, f-block, and main group metals as well being common substituents in a large number of organic compounds. Most frequently they lie at the periphery of molecules. The resultant steric accessibility has the potential for halogenated compounds to be attractive targets for use in supramolecular chemistry and crystal engineering wherein the halogen atoms are directly involved in intermolecular interactions. Indeed, interest in packing arrangements of halogenated compounds goes back many years to what Schmidt called the chloro effect, wherein the presence of chloro substituents on aromatic compounds frequently resulted in stacking arrangements with a resultant short (*ca* 4 Å) crystallographic axis. (Cohen *et al.*, 1964; Zordan *et al.*, 2005; Cohen *et al.*, 1964; Desiraju, 1989; Zhang, Li *et al.*, 2007). Herein, we chose *L*H_2_ as ligand system, and obtained a new mononuclear copper complex [Cu(*L*)(C_3_H_7_NO)] (1).

The title compound, (I), is a chiral Cu^II^ complex containing a dimethylformamide and a chiral ligand constructed from 3,5-Dichloro-2-hydroxy-benzaldehyde and 2-Amino-3-(4-hydroxy-phenyl)-propionic acid. The asymmetric unit of (I) shows a complex consisting of one Cu^II^ atom, one *L*^2-^ ligand and one dimethylformamide (Fig. 1). The Cu atom is coordinated by two oxygen atoms and one N atom from one tridentate *L*^2-^ ligand, to yield a slightly distorted planar geometry with bond lengths Cu1—O1, Cu1—O4, Cu1—O5 and Cu1—N1 1.931 (5), 1.875 (5), 1.954 (4) and 1.934 (5) Å, respectively; and bond angles (*cis*-angles are in the range of 84.5 (2)–91.9 (2) °, but all *trans*-angles are 169.0 (2)–174.3 (2) °) (Table 2).

As expected, all other bond distances and angles are within normal range. The structure forms a one-dimensional chain (Fig. 2) through weak coordination bonds (Cu1—O2^i^, 3.080 (1) Å, Cu1—Cl1^ii^, 3.269 (1) Å, symmetry codes: i: 1 + *x*, *y*, *z*; ii: -1 + *x*, *y*, *z*) and a three-dimensional network *via* weak hydrogen bonds: (O3—H3···O2^i^, 2.661 (7) Å, symmetry codes: i: 1 - *x*, 1/2 + *y*, 3/2 - *z*) and C—H···O hydrogen bond (C17—H17···O4, 2.743 (8) Å, C18—H18B···O1^ii^, 2.536 (4) Å, symmetry code: ii: 1/2 + *x*,-1/2 - *y*,2 - *z*) (Fig. 3).

### Experimental

3,5-Dichloro-2-hydroxy-benzaldehyde(0.382 g, 2.0 mmol) and 2-Amino-3-(4-hydroxy-phenyl)-propionic acid (0.3624 g, 2.0 mmol) were dissolved in 10 ml absolute methanol. The mixture was stirred for 1 h at room temperature to yield a yellow solution. To this was added a solution of CuSO_4_.5H_2_O (0.5 g, 2 mmol) in a mixture of 2 ml DMF and 10 ml me thanol. The mixture was refluxed for 1 h, and then the blue solution was filtered. Blue single crystals suitable for X-ray analysis were obtained by slow evaporation of the above filtrate at room temperature. Yield: 89.6% (based on Copper). Elemental analysis for [Cu(C_16_H_11_Cl_2_NO_4_)(C_3_H_7_NO)] calculated: C 46.69, H 3.71, N 5.73%; found: C 46.61, H 3.84, N 5.67%.

### Refinement

All hydrogen atoms were positioned geometrically and refined with a riding model, with distances 0.96 (CH_3_) or 0.93 Å (aromatic rings), and with *U*_iso_(H) = 1.2 *U*_eq_(aromatic ring) or *U*_iso_(H) = 1.5 *U*_eq_(CH_3_), O—H distance: 0.82 Å with *U*_iso_(H) = 1.5 *U*_eq_(O).

### Crystal data

**Table:**

[Cu(C_16_H_11_Cl_2_NO_4_)(C_3_H_7_NO)]	*F*_000_ = 996
*M**_r_* = 488.79	*D*_x_ = 1.560 Mg m^−^^3^
Orthorhombic, *P*2_1_2_1_2_1_	Mo *K*α radiation λ = 0.71073 Å
Hall symbol: P 2ac 2ab	Cell parameters from 4089 reflections
*a* = 5.8646 (16) Å	θ = 2.3–23.8º
*b* = 13.220 (2) Å	µ = 1.34 mm^−^^1^
*c* = 26.850 (3) Å	*T* = 298 (2) K
*V* = 2081.7 (7) Å^3^	Prism, blue
*Z* = 4	0.48 × 0.20 × 0.18 mm

### Data collection

**Table:**

Bruker SMART CCD area-detector diffractometer	3638 independent reflections
Radiation source: fine-focus sealed tube	2915 reflections with *I* > 2σ(*I*)
Monochromator: graphite	*R*_int_ = 0.098
*T* = 298(2) K	θ_max_ = 25.0º
φ and ω scans	θ_min_ = 1.5º
Absorption correction: multi-scan(SADABS; Sheldrick, 1996)	*h* = −6→6
*T*_min_ = 0.532, *T*_max_ = 0.786	*k* = −15→12
10650 measured reflections	*l* = −31→31

### Refinement

**Table:**

Refinement on *F*^2^	Hydrogen site location: inferred from neighbouring sites
Least-squares matrix: full	H-atom parameters constrained
*R*[*F*^2^ > 2σ(*F*^2^)] = 0.061	*w* = 1/[σ^2^(*F*_o_^2^) + (0.0751*P*)^2^] where *P* = (*F*_o_^2^ + 2*F*_c_^2^)/3
*wR*(*F*^2^) = 0.148	(Δ/σ)_max_ < 0.001
*S* = 1.04	Δρ_max_ = 0.50 e Å^−^^3^
3638 reflections	Δρ_min_ = −0.54 e Å^−^^3^
262 parameters	Extinction correction: none
Primary atom site location: structure-invariant direct methods	Absolute structure: Flack (1983), with 1505 Friedel pairs
Secondary atom site location: difference Fourier map	Flack parameter: 0.04 (3)

### Special details

**Table:**

Geometry. All e.s.d.'s (except the e.s.d. in the dihedral angle between two l.s. planes) are estimated using the full covariance matrix. The cell e.s.d.'s are taken into account individually in the estimation of e.s.d.'s in distances, angles and torsion angles; correlations between e.s.d.'s in cell parameters are only used when they are defined by crystal symmetry. An approximate (isotropic) treatment of cell e.s.d.'s is used for estimating e.s.d.'s involving l.s. planes.
Refinement. Refinement of *F*^2^ against ALL reflections. The weighted *R*-factor *wR* and goodness of fit *S* are based on *F*^2^, conventional *R*-factors *R* are based on *F*, with *F* set to zero for negative *F*^2^. The threshold expression of *F*^2^ > σ(*F*^2^) is used only for calculating *R*-factors(gt) *etc*. and is not relevant to the choice of reflections for refinement. *R*-factors based on *F*^2^ are statistically about twice as large as those based on *F*, and *R*- factors based on ALL data will be even larger.

### Fractional atomic coordinates and isotropic or equivalent isotropic displacement parameters (Å^2^)

**Table:**

	*x*	*y*	*z*	*U*_iso_*/*U*_eq_	
Cu1	0.87095 (14)	0.01109 (5)	0.92554 (2)	0.0398 (2)	
Cl1	1.5356 (3)	0.15751 (14)	0.99080 (6)	0.0542 (5)	
Cl2	1.5314 (5)	0.4447 (2)	0.84884 (10)	0.1123 (10)	
N1	0.7954 (8)	0.0937 (4)	0.86843 (16)	0.0341 (12)	
N2	1.2035 (10)	−0.1364 (5)	1.03525 (19)	0.0502 (15)	
O1	0.5867 (8)	−0.0565 (3)	0.91109 (13)	0.0447 (11)	
O2	0.2837 (8)	−0.0471 (4)	0.86065 (16)	0.0507 (12)	
O3	0.9847 (9)	0.2916 (4)	0.64815 (17)	0.0663 (15)	
H3	0.8857	0.3335	0.6420	0.099*	
O4	1.1115 (8)	0.0946 (3)	0.94609 (13)	0.0426 (10)	
O5	0.9417 (9)	−0.0841 (4)	0.97899 (16)	0.0566 (14)	
C1	0.4783 (12)	−0.0227 (4)	0.8738 (2)	0.0371 (14)	
C2	0.5969 (10)	0.0579 (4)	0.84153 (18)	0.0335 (13)	
H2	0.4921	0.1145	0.8357	0.040*	
C3	0.6629 (11)	0.0092 (4)	0.79128 (18)	0.0413 (15)	
H3A	0.7794	−0.0416	0.7971	0.050*	
H3B	0.5305	−0.0249	0.7776	0.050*	
C4	0.7505 (11)	0.0844 (5)	0.7537 (2)	0.0390 (15)	
C5	0.6177 (12)	0.1646 (5)	0.7384 (2)	0.0470 (16)	
H5	0.4740	0.1726	0.7525	0.056*	
C6	0.6887 (12)	0.2333 (5)	0.7032 (2)	0.0478 (18)	
H6	0.5919	0.2850	0.6930	0.057*	
C7	0.9055 (11)	0.2249 (5)	0.6832 (2)	0.0436 (16)	
C8	1.0373 (13)	0.1453 (5)	0.6970 (2)	0.0552 (18)	
H8	1.1793	0.1369	0.6822	0.066*	
C9	0.9657 (12)	0.0762 (5)	0.7326 (2)	0.0477 (17)	
H9	1.0625	0.0241	0.7424	0.057*	
C10	0.8988 (10)	0.1759 (4)	0.85633 (19)	0.0335 (13)	
H10	0.8373	0.2130	0.8301	0.040*	
C11	1.1005 (11)	0.2157 (4)	0.87951 (19)	0.0367 (14)	
C12	1.1995 (9)	0.1712 (4)	0.9226 (2)	0.0346 (13)	
C13	1.4061 (11)	0.2142 (5)	0.93978 (19)	0.0372 (14)	
C14	1.5016 (12)	0.2978 (5)	0.9188 (2)	0.0527 (18)	
H14	1.6334	0.3259	0.9322	0.063*	
C15	1.4005 (14)	0.3402 (5)	0.8774 (3)	0.062 (2)	
C16	1.2032 (12)	0.3018 (5)	0.8584 (3)	0.0508 (18)	
H16	1.1354	0.3329	0.8311	0.061*	
C17	1.1375 (15)	−0.0875 (5)	0.9947 (3)	0.0558 (18)	
H17	1.2486	−0.0531	0.9767	0.067*	
C18	1.0416 (14)	−0.1899 (7)	1.0655 (3)	0.073 (2)	
H18A	1.0066	−0.1505	1.0945	0.110*	
H18B	1.1053	−0.2537	1.0754	0.110*	
H18C	0.9048	−0.2013	1.0467	0.110*	
C19	1.4387 (14)	−0.1337 (7)	1.0521 (3)	0.078 (3)	
H19A	1.5335	−0.1067	1.0261	0.118*	
H19B	1.4881	−0.2010	1.0601	0.118*	
H19C	1.4502	−0.0917	1.0811	0.118*	

### Atomic displacement parameters (Å^2^)

**Table:**

	*U*^11^	*U*^22^	*U*^33^	*U*^12^	*U*^13^	*U*^23^
Cu1	0.0472 (4)	0.0363 (4)	0.0360 (3)	−0.0017 (4)	−0.0031 (3)	0.0056 (3)
Cl1	0.0510 (10)	0.0635 (11)	0.0481 (8)	0.0039 (9)	−0.0144 (7)	−0.0136 (8)
Cl2	0.117 (2)	0.0863 (18)	0.133 (2)	−0.0606 (17)	−0.0291 (18)	0.0441 (15)
N1	0.037 (3)	0.031 (3)	0.034 (2)	0.009 (2)	0.001 (2)	−0.002 (2)
N2	0.045 (3)	0.056 (4)	0.050 (3)	0.007 (3)	−0.001 (3)	0.022 (3)
O1	0.056 (3)	0.039 (2)	0.039 (2)	−0.010 (2)	0.001 (2)	0.0045 (17)
O2	0.043 (3)	0.048 (3)	0.060 (3)	−0.008 (2)	0.004 (2)	−0.009 (2)
O3	0.070 (4)	0.058 (3)	0.071 (3)	0.012 (3)	0.034 (3)	0.015 (3)
O4	0.047 (3)	0.045 (2)	0.0357 (19)	0.005 (2)	−0.003 (2)	−0.0015 (18)
O5	0.060 (4)	0.055 (3)	0.055 (3)	0.002 (3)	−0.006 (2)	0.024 (2)
C1	0.043 (4)	0.027 (3)	0.041 (3)	0.003 (3)	0.001 (3)	−0.007 (3)
C2	0.024 (3)	0.040 (3)	0.037 (3)	0.000 (3)	−0.005 (3)	−0.004 (2)
C3	0.049 (4)	0.039 (3)	0.036 (3)	−0.003 (4)	−0.003 (3)	−0.005 (3)
C4	0.043 (4)	0.040 (4)	0.034 (3)	0.005 (3)	−0.007 (3)	−0.003 (3)
C5	0.036 (3)	0.067 (4)	0.038 (3)	0.007 (4)	0.003 (3)	0.005 (3)
C6	0.046 (4)	0.050 (4)	0.047 (4)	0.015 (3)	0.003 (3)	0.007 (3)
C7	0.043 (4)	0.051 (4)	0.037 (3)	0.004 (4)	0.005 (3)	0.001 (3)
C8	0.048 (4)	0.062 (5)	0.056 (4)	0.010 (4)	0.014 (3)	0.007 (3)
C9	0.041 (4)	0.046 (4)	0.056 (4)	0.009 (3)	0.007 (3)	−0.006 (3)
C10	0.032 (3)	0.034 (3)	0.034 (3)	−0.001 (3)	0.000 (3)	0.001 (2)
C11	0.038 (4)	0.035 (3)	0.037 (3)	0.001 (3)	0.004 (3)	−0.001 (2)
C12	0.027 (3)	0.036 (3)	0.041 (3)	0.005 (3)	0.006 (3)	−0.017 (3)
C13	0.035 (4)	0.045 (4)	0.032 (3)	0.003 (3)	0.001 (3)	−0.013 (2)
C14	0.045 (4)	0.052 (4)	0.061 (4)	−0.010 (4)	−0.005 (4)	−0.015 (3)
C15	0.060 (5)	0.052 (4)	0.073 (5)	−0.013 (4)	−0.001 (4)	0.009 (4)
C16	0.050 (4)	0.038 (4)	0.064 (4)	−0.012 (3)	−0.002 (4)	0.014 (3)
C17	0.055 (5)	0.048 (4)	0.065 (4)	0.009 (4)	0.001 (4)	0.021 (3)
C18	0.063 (5)	0.093 (6)	0.064 (5)	0.001 (5)	0.003 (4)	0.038 (4)
C19	0.055 (5)	0.092 (7)	0.088 (5)	0.003 (5)	−0.017 (4)	0.037 (5)

### Geometric parameters (Å, °)

**Table:**

Cu1—O4	1.875 (5)	C5—C6	1.374 (9)
Cu1—O1	1.931 (5)	C5—H5	0.9300
Cu1—N1	1.934 (5)	C6—C7	1.385 (9)
Cu1—O5	1.954 (4)	C6—H6	0.9300
Cl1—C13	1.736 (6)	C7—C8	1.357 (9)
Cl2—C15	1.756 (7)	C8—C9	1.387 (9)
N1—C10	1.286 (7)	C8—H8	0.9300
N1—C2	1.449 (7)	C9—H9	0.9300
N2—C17	1.324 (8)	C10—C11	1.437 (8)
N2—C18	1.435 (9)	C10—H10	0.9300
N2—C19	1.452 (9)	C11—C16	1.407 (8)
O1—C1	1.267 (7)	C11—C12	1.423 (8)
O2—C1	1.238 (7)	C12—C13	1.416 (8)
O3—C7	1.371 (7)	C13—C14	1.361 (9)
O3—H3	0.8200	C14—C15	1.379 (10)
O4—C12	1.299 (7)	C14—H14	0.9300
O5—C17	1.224 (9)	C15—C16	1.362 (10)
C1—C2	1.540 (8)	C16—H16	0.9300
C2—C3	1.545 (7)	C17—H17	0.9300
C2—H2	0.9800	C18—H18A	0.9600
C3—C4	1.508 (8)	C18—H18B	0.9600
C3—H3A	0.9700	C18—H18C	0.9600
C3—H3B	0.9700	C19—H19A	0.9600
C4—C5	1.378 (9)	C19—H19B	0.9600
C4—C9	1.387 (9)	C19—H19C	0.9600
			
O4—Cu1—O1	169.0 (2)	C7—C8—C9	121.8 (7)
O4—Cu1—N1	94.2 (2)	C7—C8—H8	119.1
O1—Cu1—N1	84.5 (2)	C9—C8—H8	119.1
O4—Cu1—O5	90.2 (2)	C4—C9—C8	120.3 (7)
O1—Cu1—O5	91.9 (2)	C4—C9—H9	119.9
N1—Cu1—O5	174.3 (2)	C8—C9—H9	119.9
C10—N1—C2	121.9 (5)	N1—C10—C11	126.1 (5)
C10—N1—Cu1	124.7 (4)	N1—C10—H10	117.0
C2—N1—Cu1	113.3 (4)	C11—C10—H10	117.0
C17—N2—C18	120.8 (6)	C16—C11—C12	119.2 (6)
C17—N2—C19	121.4 (7)	C16—C11—C10	118.3 (5)
C18—N2—C19	117.7 (6)	C12—C11—C10	122.5 (5)
C1—O1—Cu1	115.4 (4)	O4—C12—C13	119.7 (5)
C7—O3—H3	109.5	O4—C12—C11	123.7 (5)
C12—O4—Cu1	128.0 (4)	C13—C12—C11	116.6 (6)
C17—O5—Cu1	118.4 (5)	C14—C13—C12	122.9 (6)
O2—C1—O1	126.6 (6)	C14—C13—Cl1	119.8 (5)
O2—C1—C2	115.8 (5)	C12—C13—Cl1	117.2 (5)
O1—C1—C2	117.6 (5)	C13—C14—C15	119.2 (7)
N1—C2—C1	107.9 (4)	C13—C14—H14	120.4
N1—C2—C3	111.7 (5)	C15—C14—H14	120.4
C1—C2—C3	108.4 (5)	C16—C15—C14	121.0 (7)
N1—C2—H2	109.6	C16—C15—Cl2	120.1 (6)
C1—C2—H2	109.6	C14—C15—Cl2	118.9 (6)
C3—C2—H2	109.6	C15—C16—C11	121.0 (7)
C4—C3—C2	113.3 (5)	C15—C16—H16	119.5
C4—C3—H3A	108.9	C11—C16—H16	119.5
C2—C3—H3A	108.9	O5—C17—N2	125.2 (7)
C4—C3—H3B	108.9	O5—C17—H17	117.4
C2—C3—H3B	108.9	N2—C17—H17	117.4
H3A—C3—H3B	107.7	N2—C18—H18A	109.5
C5—C4—C9	117.0 (6)	N2—C18—H18B	109.5
C5—C4—C3	121.0 (6)	H18A—C18—H18B	109.5
C9—C4—C3	122.1 (6)	N2—C18—H18C	109.5
C6—C5—C4	122.8 (7)	H18A—C18—H18C	109.5
C6—C5—H5	118.6	H18B—C18—H18C	109.5
C4—C5—H5	118.6	N2—C19—H19A	109.5
C5—C6—C7	119.4 (6)	N2—C19—H19B	109.5
C5—C6—H6	120.3	H19A—C19—H19B	109.5
C7—C6—H6	120.3	N2—C19—H19C	109.5
C8—C7—O3	119.5 (6)	H19A—C19—H19C	109.5
C8—C7—C6	118.6 (6)	H19B—C19—H19C	109.5
O3—C7—C6	121.7 (6)		
			
O4—Cu1—N1—C10	−0.5 (5)	O3—C7—C8—C9	179.5 (6)
O1—Cu1—N1—C10	168.5 (5)	C6—C7—C8—C9	−3.9 (11)
O4—Cu1—N1—C2	−176.4 (4)	C5—C4—C9—C8	−1.5 (10)
O1—Cu1—N1—C2	−7.4 (3)	C3—C4—C9—C8	178.0 (6)
O4—Cu1—O1—C1	83.8 (9)	C7—C8—C9—C4	2.9 (11)
N1—Cu1—O1—C1	0.1 (4)	C2—N1—C10—C11	−177.8 (5)
O5—Cu1—O1—C1	−175.4 (4)	Cu1—N1—C10—C11	6.6 (8)
O1—Cu1—O4—C12	−90.9 (9)	N1—C10—C11—C16	174.1 (6)
N1—Cu1—O4—C12	−8.1 (5)	N1—C10—C11—C12	−5.7 (9)
O5—Cu1—O4—C12	168.2 (5)	Cu1—O4—C12—C13	−168.8 (4)
O4—Cu1—O5—C17	−26.4 (6)	Cu1—O4—C12—C11	10.8 (8)
O1—Cu1—O5—C17	164.4 (6)	C16—C11—C12—O4	176.7 (5)
Cu1—O1—C1—O2	−172.3 (5)	C10—C11—C12—O4	−3.5 (8)
Cu1—O1—C1—C2	7.0 (6)	C16—C11—C12—C13	−3.7 (8)
C10—N1—C2—C1	−164.2 (5)	C10—C11—C12—C13	176.1 (5)
Cu1—N1—C2—C1	11.9 (5)	O4—C12—C13—C14	−176.3 (5)
C10—N1—C2—C3	76.7 (7)	C11—C12—C13—C14	4.1 (8)
Cu1—N1—C2—C3	−107.2 (4)	O4—C12—C13—Cl1	2.3 (7)
O2—C1—C2—N1	166.9 (5)	C11—C12—C13—Cl1	−177.3 (4)
O1—C1—C2—N1	−12.5 (7)	C12—C13—C14—C15	−3.4 (10)
O2—C1—C2—C3	−71.9 (6)	Cl1—C13—C14—C15	178.1 (5)
O1—C1—C2—C3	108.7 (5)	C13—C14—C15—C16	2.2 (11)
N1—C2—C3—C4	−69.3 (6)	C13—C14—C15—Cl2	−177.3 (5)
C1—C2—C3—C4	171.9 (5)	C14—C15—C16—C11	−2.0 (11)
C2—C3—C4—C5	−59.0 (7)	Cl2—C15—C16—C11	177.5 (6)
C2—C3—C4—C9	121.5 (6)	C12—C11—C16—C15	2.8 (10)
C9—C4—C5—C6	1.3 (10)	C10—C11—C16—C15	−177.0 (6)
C3—C4—C5—C6	−178.2 (6)	Cu1—O5—C17—N2	169.3 (5)
C4—C5—C6—C7	−2.4 (10)	C18—N2—C17—O5	−1.1 (12)
C5—C6—C7—C8	3.6 (10)	C19—N2—C17—O5	−178.0 (8)
C5—C6—C7—O3	−179.8 (6)		

### Hydrogen-bond geometry (Å, °)

**Table:**

*D*—H···*A*	*D*—H	H···*A*	*D*···*A*	*D*—H···*A*
O3—H3···O2^i^	0.82	1.87	2.661 (7)	163
C17—H17···O4	0.93	2.27	2.743 (8)	111
C18—H18B···O1^ii^	0.96	2.54	3.421 (10)	150

Symmetry codes: (i) −*x*+1, *y*+1/2, −*z*+3/2; (ii) *x*+1/2, −*y*−1/2, −*z*+2.
